# Navigation and non-navigation CT scan of the sinuses: comparison of the effective doses of radiation in children and adults

**DOI:** 10.1186/s40463-021-00541-x

**Published:** 2021-11-19

**Authors:** Noémie Villemure-Poliquin, Mario Chrétien, Jacques E. Leclerc

**Affiliations:** 1 Département d’ophtalmologie et d’oto-rhino-laryngologie – chirurgie cervico-faciale, Faculté de Médecine, Université Laval, Québec, QC Canada; 2 Service de Physique Médicale et de Radioprotection, CHU de Québec – Université Laval, Pavillon Enfant-Jésus, 1401 18e Rue, Québec, G1J 1Z4 Canada

**Keywords:** Sinusitis, Paediatric sinusitis, Polyps, Cystic fibrosis, Nasosinusal tumors, Sinus surgery, Endoscopic sinus surgery, Ionizing radiation, Effective dose, 3D navigation, Neuronavigation

## Abstract

**Background:**

The advent of 3D navigation imaging has opened new borders to the endoscopic surgical approaches of naso-sinusal inflammatory and neoplastic disease. This technology has gained in popularity among otolaryngologists for endoscopic sinus and skull base surgeries in both adults and children. However, the increased tissue radiation required for data acquisition associated with 3D navigation protocols CT scans is a source of concern because of its potential health hazards. We aimed to compare the effective doses of radiation between 3D navigation protocols and standard protocols for sinus computed tomography (CT) scans for both the adult and pediatric population.

**Methods:**

We performed a retrospective cohort study through electronic chart review of patients undergoing sinus CT scans (standard and 3D navigation protocols) from May 2019 to December 2019 using a Siemens Drive (VA62A) CT scanner. The effective dose of radiation was calculated in mSv for all exams. Average irradiation doses were compared using a Student’s *T*-Test or a Kruskall–Wallis test when appropriate.

**Results:**

A total of 115 CT scans were selected for analysis, of which 47 were standard protocols and 68 were 3D navigation protocols CT scans. Among these, 31 exams were performed on children and 84 exams on adults. For the total population, mean effective dose in the non-navigation CT scans was 0.37 mSv (SD: 0.16, N = 47) and mean effective dose in the 3D navigation sinus CT group was 2.33 mSv (SD: 0.45, N = 68). The mean difference between the two groups was statistically significant 1.97 mSv (CI 95% − 2.1 to − 1.83; *P* < 0.0001). There was a sixfold increase in radiation with utilization of 3D navigation protocols. The ratio was identical when the pediatric as well as the adult subset of patients were analyzed.

**Conclusion:**

In our center, utilization of 3D navigation sinus CT protocols significantly increases radiation exposure. Otolaryngologists should be aware of this significant increase and should attempt to decrease the radiation exposure of their patients by limiting unnecessary scan orders and by evaluating 3D acquisition protocols locally with radiation physicists.

**Level of evidence**: Level IV.

**Graphical Abstract:**

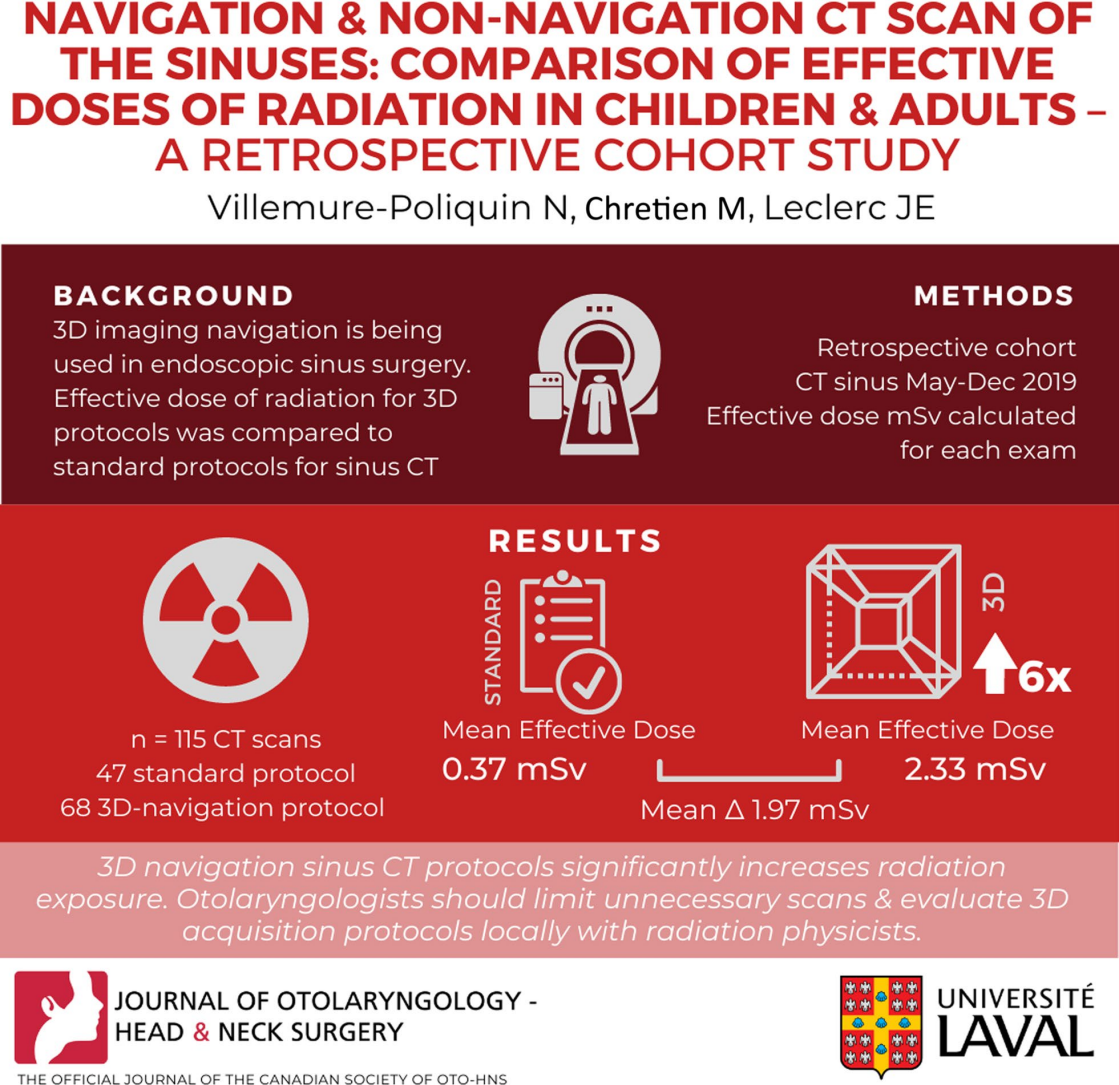

## Background

The advent of 3D navigation imaging has opened new borders to the endoscopic surgical approaches of naso-sinusal inflammatory and neoplastic disease [1–8]. The outstanding precision obtained from the navigation systems increases the safety level of the endoscopic procedures [9–11]. The use of navigation systems has recently gained in popularity among the pediatric population, particularly in the fields of neurosurgery and otolaryngology. The use of this technology has been described for several pediatric surgeries, including intracerebral tumor resections, skull base tumor resections, choanal atresia cures as well as endoscopic sinus surgeries. 3D navigation can be particularly useful in a very young population, for whom all the anatomical structures are smaller and in whom there is an increased risk of complications [5, 10]. However, the increased tissue radiation required for data acquisition associated with navigation (3D) CT scans is a source of concern in the medical literature because of its potential health hazards [12]. Radiation-induced cancers are certainly the most feared complications. This risk is notably greater in children, due to their higher radiosensitivity than adults and to a longer lifespan after radiation exposure [13–15]. Several strategies for reducing radiation doses have been studied. They include a modification in acquisition parameters, the use of iterative reconstructive techniques, the use of eye lens shielding and the adoption of cone beam technologies [16–18]. As stated by Hoxworth and Lal [17], the best way to decrease radiation exposure is to avoid unnecessary scanner examinations. To our knowledge, no study has quantified the difference of effective dose of radiation caused by the use of a 3D navigation acquisition protocol compared to a standard protocol specifically for CT scans of the sinuses, either in children or adult patients. The aim of our study is therefore to compare the effective radiation doses associated with CT scans of the sinuses with a 3D navigation protocol to those with a standard protocol in our tertiary referral center. The objectives of this study were threefold: (1) To survey the prescription habits of 3D navigation sinus CT in our center; (2) To compare the effective doses of radiation between navigation and non-navigation CT scans of the sinuses in the pediatric and adult population and (3) To compare the effective doses of radiation between children and adults for a similar navigation protocol.

### ABC of ionizing radiation

Ionizing radiation includes gamma rays, X-rays and the higher ultraviolet part of the electromagnetic spectrum as opposed to the lower ultraviolet part of the spectrum, visible light, infrared, microwaves and radio waves which are examples of non-ionizing radiation. As previously stated, exposure to ionizing radiation has been shown to increase the incidence of neoplasia. There is evidence suggesting that radiation exposure in childhood is associated with a higher risk of developing various types of cancers later in life including leukemia, thyroid, skin, breast and brain cancer [19–21].

Three dosimetry quantities have been defined to measure radiation: the absorbed dose, the equivalent dose and the effective dose. The absorbed dose corresponds to the amount of energy deposited in a substance (e.g., human tissue). The absorbed dose is measured in a unit called the gray (Gy). A dose of one gray is equivalent to a unit of energy (joule) deposited in a kilogram of a substance [22]. The equivalent dose considers the damaging properties of the various types of radiation and is based on the absorbed dose by a tissue or an organ. This weighted absorbed quantity is expressed in a unit called the sievert (Sv). Finally, the effective dose is a measure of the total detriment or risk, due to exposure to ionizing radiation. The effective dose calculation combines the absorbed dose to the whole body, the relative harm level of the radiation as well as the specific sensitivity of each organ to ionizing radiation. If the exposure to different organs or tissues is not uniform, the concept of effective dose is used. The most significant dose quantity for patients is certainly the effective dose, as it allows for comparison and evaluation of long-term risk [23, 24]. The basic idea is to express the risk from the exposure to a single organ or tissue in terms of the equivalent risk from an exposure to the whole body. The unit of effective dose is the sievert. The three dosimetry quantities are protection quantities defined by The International Commission on Radiological Protection (ICRP) [25].

**Fig. 1:**
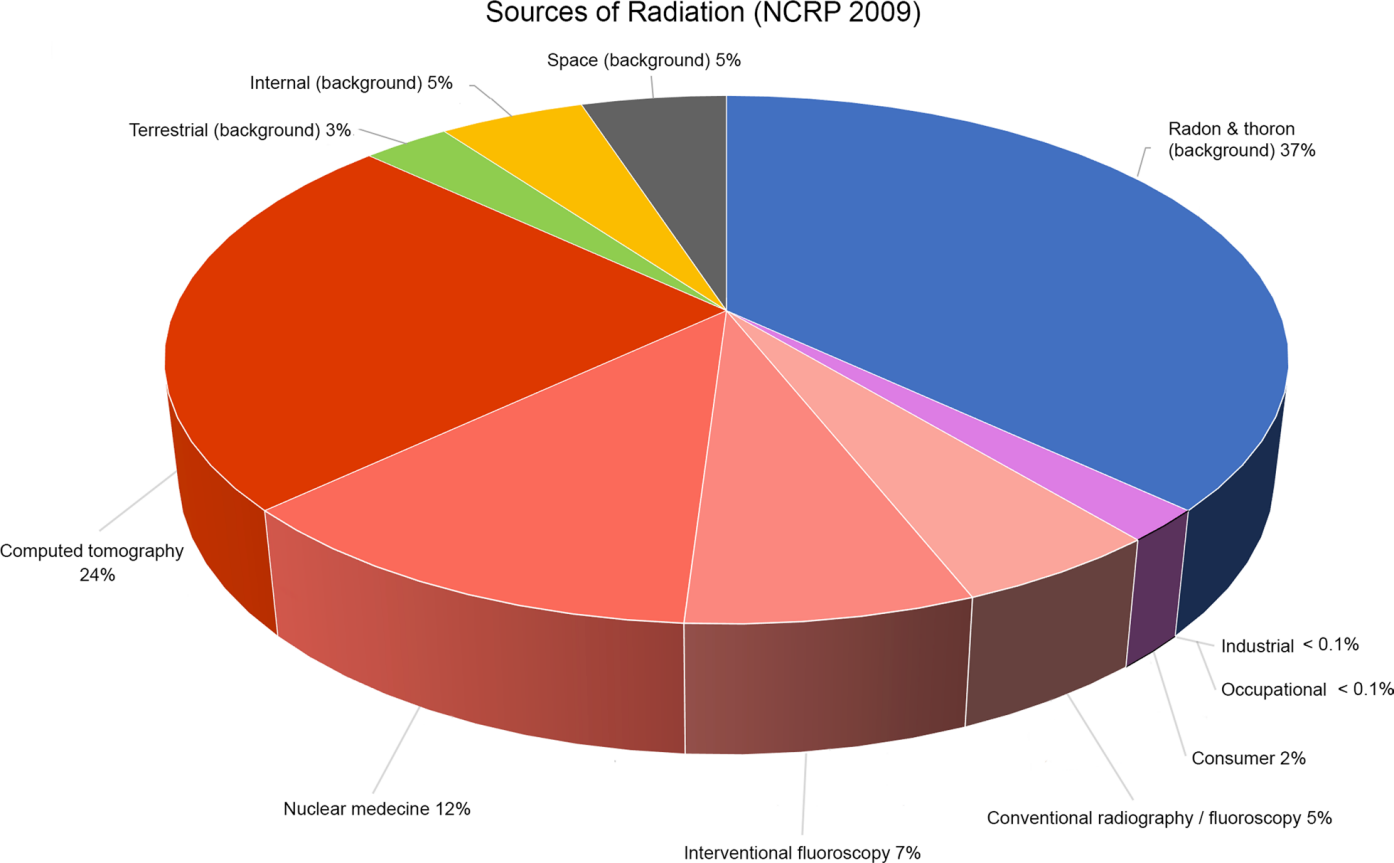
Sources of ionizing radiation (NCRP Report 160, 2009)

**Fig. 2:**
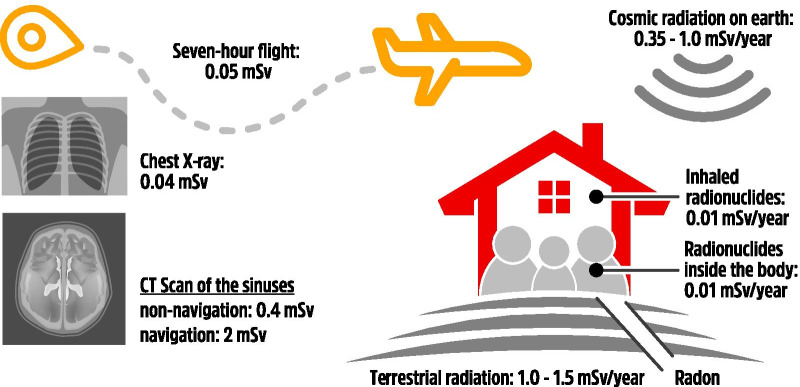
Effective doses of radiation (mSv) from different sources (total 6.2 mSv/year per individual—NCRP Report 160 (2009))

In 2009, the National Council on Radiation Protection and Measurements (NCRP) has published Report 160: Ionizing Radiation Exposure of the Population of the United States [26]. This report evaluated the doses to the U.S. population from all sources of ionizing radiation in 2006. The total effective dose from all sources was estimated at 6.2 mSv/year per individual. The average annual effective dose results from ubiquitous natural background (50%) and medical exposure of patients (48%), estimated to be approximately 3 mSv for each [26]. The main sources of ionizing radiation were natural background radon and thoron gaz (37%) and medical exposure with CT (24%) and nuclear medicine (12%) (Figs. 1, 2). Considering only the natural background dose, the added amounts of radiation over childhood and a lifetime period of 80 years are respectively 50 mSv and 250 mSv. The previous NCRP Report 93 (1987) [27] detailed the exposure of the US population during the early 1980s. From ubiquitous natural background radiation, the average individual effective dose was estimated to be similar. However, the average yearly dose of ionizing radiation from medical exposures was about 0.5 mSv per person in the USA during the 1980s; by 2006, it had increased to 3.0 mSv per person [14]. The increase is primarily due to the use of CT scans (responsible for ∼1/2 the 2006 medical dose), nuclear medicine (∼1/4 the dose mainly due to nuclear cardiology) and interventional fluoroscopy [26, 28].

## Materials and methods

This study was conducted at the Centre Hospitalier Universitaire de Québec – Université Laval after approval from the Board of Ethics. From May 2019 to December 2019, we performed an electronic chart review of all patients who underwent both navigation and non-navigation CT scans of the sinuses. Patients could only be classified in one of the two groups (navigation vs non-navigation). For example, if a patient had had both standard and 3D navigation sinus CT, he was automatically classified in the navigation group. We collected data for both the pediatric and adult population. The scanner used for all CT exams was a Siemens Drive (Syngo CT VA62A) and data acquisition for the 3D navigation was based on the current recommendations from Stryker Co. (Table 1). Data relative to the CT parameters, such as kilovoltage (Kv), milliampere-seconds (mAs) and volumetric computed tomography dose index (CTDIv) were collected and analyzed by a medical radiation physicist (M.C.).

**Table 1:** Comparison of parameters of standard and navigation sinus CT scan protocols for 3 navigation systems

	Stryker		Brainlab		Medtronic	
	Non navigation	Navigation	Non navigation	Navigation	Non navigation	Navigation
Field of view	Same during scan	Same during scan	Same during scan	Same during scan	Same during scan	Same during scan
Slice thickness	1 mm or 1.25 mm	1 mm or 1.25 mm	≤ 3 mm	≤ 2 mm	< 3 mm	1 mm
Contiguous scan	Yes	Yes	No: slice thickness < slice distance	Yes	Yes	Yes
Overlapping	No	No	No	No	No	No
Pixel/matrix	Square	Square	Square	Square	Square	Square
Pixel/matrix: minimum size	256 × 256	256 × 256	256 × 256	256 × 256	256 × 256	256 × 256
Pixel/matrix: maximum size	512 × 512	512 × 512	512 × 512	512 × 512	512 × 512	512 × 512

The CTDIv is the primary dose measurement concept in CT scanning. The CTDI represents the average absorbed dose in mGy [27]. It is calculated by the scanner based on the radiation output for the particular scan which would be absorbed by a polymethylmethacrylate (PMMA, e.g., acrylic or Lucite™) cylinder model. The head model is a 14-cm length cylinder with a 16 cm diameter. It is typically referred to as the head CTDI phantom. It is not a dose administered to a specific patient, but it is meant to be a comparison metric for different scans. The dose-linear product (DLP) is calculated by multiplying the CTDI-vol (which is obtained for a centimeter) by the number of centimeters scanned. Its result is presented in units of mGy-cm.

The effective radiation of each scan was calculated in millisieverts (mSv), based on dose-length product (DLP) reported by the CT scanner and using a conversion factor obtained from Table 3 of the Report No. 96 of the American Association of Physicists in Medicine [29]. The use of DLP to estimate the effective dose appears to be a reasonably robust method for estimating the effective dose. The values of Table 3 [29] are for adults with standard physique and pediatric patients of various ages over various body regions. Conversion factor for adult head and neck and pediatric patients assume the use of the head CT dose phantom (16 cm). The conversion factors used for calculations were 0.013 (birth), 0.0085 (1-year-old), 0.0057 (5-year-old), 0.0042 (10-year-old) and 0.0031 (adult) (Table 3, [29]), [30].

The rest of the data collection was performed by the other co-authors (J.L. and N.V.P). The indications and the specialty of the physician who ordered the CT scan were obtained for all patients. The number of CTs performed for the same episode of care as well as the presence of a surgical intervention after CT were collected for the 3D navigation protocol group. Student’s *t*-test was used to compare the navigation to the non-navigation pediatric and adult effective dose values. It was also used to compare the pediatric versus the adult effective dose results. The significance level was set at *P* ≤ 0.05. The Kruskal–Wallis test was used to confirm the results because of the small size of the cohort. All analyses were performed with SAS university 3.8 software.

## Results

### Population description

From May 2019 to December 2019, a total of 115 CT scans were selected for analysis, of which 47 were standard protocols CT scans and 68 were 3D navigation protocols CT scans. A total of 31 children and 84 adults were included in the analysis. In the pediatric cohort, 14 patients (45%, mean age = 10.9 years old) underwent standard protocol non-navigation sinus CT scans and 17 underwent 3D navigation protocol sinus CT scans (55%, mean age = 9.8 years old). Demographic data of both pediatric and adult cohorts can be found in Table 2. In Table 3, the list of indications for the scans and the prescribing specialties are shown for both the pediatric and adult cohort. In the children 3D navigation group, the majority of patients had had only one CT examination for the same indication (7/11). Four patients had a standard CT before the 3D navigation sinus CT scan. Of the 17 patients, 11 underwent surgical intervention after the navigation CT scan. In the other 6 patients, the CT scan did not yield indications for surgical intervention. In both children and adults, most of the navigation CT scans were prescribed by otolaryngologists.

**Table 2:** Demographic data

		Standard	Navigation	*P* value
Pediatric	N	14	17	
	Age (SD)	10.9 (4.9)	9.8 (4.5)	0.52
	Weight (SD)	39.3 (18.1)	34.5 (16.5)	0.44
Adults	N	33	51	
	Age (SD)	51.9 (18.6)	50.1 (13.9)	0.63

**Table 3:** Standard and navigation CT scans: indications and prescribing physicians

Children standard CT scans	14	Children navigation CT scans	17
*Indications*			
Persistent sinusitis symptoms	6	Suspected or confirmed chronic sinusitis	9
Recurrent sinusitis	2	Complicated acute sinusitis	3
Confirmation fortuitous finding another exam	2	Choanal atresia	3
Febrile neutropenia	1	Mass lesion: craniopharyngioma and maxillary myxoma	2
Hyposmia	1		
Fungal pulmonary disease	1		
Choanal atresia follow-up	1		
*Physician*			
Otolaryngologist	6	Otolaryngologist	15
Pediatrician	5	Ophtalmologist	1
General practitioner	3	Pediatrician	1

Adults standard CT scans	33	Adults navigation CT scans	51
*Indications*			
Suspected or confirmed chronic sinusitis	22	Suspected or confirmed chronic sinusitis	42
Recurrent epistaxis	2	Recurrent sinusitis	6
Epiphora	2	Headache	1
Recurrent sinusitis	1	Hyposmia	1
Facial pain	1	Recurrent epistaxis	1
Hyposmia	1		
Granulomatosis with polyangiitis disease	1		
Headache	1		
Eosinophilia	1		
Nasal cancer	1		
*Physicians*			
General practitioner	14	Otolaryngologist	51
Otolaryngologist	9		
Internal medecine	7		
Ophtalmologist	2		
Infectious disease	1		

In our adult cohort, we found 33 (40%) non-navigation and 51 (60%) 3D navigation CT scans. The latter were prescribed exclusively by otolaryngologists. Of the 51 patients, only 25 underwent or had an indication for sinus surgery following 3D navigation CT scanning. In the other 26 patients imaging results did not reach criteria for sinus surgery. In the latter group, 21 patients had one or more sinus CT for the same indication. The majority were standard sinus CT protocols (14 patients) and less often, patients had another 3D navigation sinus CT scan (7 patients).

### Comparison of irradiation doses

The scanner protocols for non-navigation and navigation scans were respectively: cut thickness 0.6 mm versus 1 mm; 120 kV versus 120 kV; 120 mAs versus 390 mAs. For the total population of effective radiation doses, the mean effective dose in the non-navigation CT group was 0.37 mSv (SD: 0.16, N = 47) and the mean effective dose in the 3D navigation sinus CT group was 2.33 mSv (SD: 0.45, N = 68). The mean difference between the two groups was statistically significant: − 1.97 mSv (CI 95% − 2.1 to − 1.83; *P* < 0.0001). Significant differences were also found when both pediatric and adult cohorts were compared separately. The mean difference of effective radiation dose was of 2.06 mSv (IC 95%: − 2.17 to − 1.96 mSv; *P* < 0.0001) for the adult cohort and was 1.68 mSv (IC 95%: − 2.07 to − 1.29 mSv; *P* < 0.0001) for the pediatric cohort. Effective radiation dose averages for the 3D navigation sinus CT scan protocols were compared between the pediatric population and the adult population. We also found a statistically significant mean difference of 0.41 mSv (IC 95%: − 0.64 to − 0.17; *P* = 0.03) between the two groups, with a higher level in adults. No difference was found between the 2 groups for the standard sinus protocol. A summary of the findings is presented in Table 4.

**Table 4:** Summary of results

		N	mSv (SD)	Difference (CI 95%)	*P* value
Total	Standard	47	0.37 (0.16)	− 1.97 (− 2.1 to − 1.83)	< 0.0001
	Navigation	68	2.33 (0.45)		
Adults	Standard	33	0.37 (0.18)	− 2.06 (− 2.17 to − 1.96)	< 0.0001
	Navigation	51	2.44 (0.26)		
Children	Standard	14	0.35 (0.11)	− 1.68 (− 2.07 to − 1.29)	< 0.0001
	Navigation	17	2.03 (0.71)		
Standard protocol	Children	14	0.35 (0.11)	− 0.02 (− 0.11 to 0.08)	0.65
	Adults	33	0.37 (0.18)		
Navigation protocol	Children	17	2.03 (0.71)	− 0.41 (− 0.64 to − 0.17)	0.03
	Adults	51	2.44 (0.26)		

## Discussion

### Head versus head and neck conversion factors

The difference between the two conversion factors (head vs head and neck) does not come from the size of the scans but from the type of tissues/organs involved. The ‘’Head’’ set of values include the brain, skin and bones. The ‘’Head and Neck’’ was used because the data acquisition for CT scans of the sinuses is done supine. The coronal cuts extend all the way down to the upper teeth to include the gums and the roots of the teeth. These cuts also include a significant portion of the parotid glands and parts of the submandibular glands. Of course, this setting normally includes the upper part of the esophagus, thyroid gland and some bone medulla. These tissues are not included in CT scans of the sinuses. The absolute value of effective radiation that we reported must be considered as the maximal possible dose. This has no impact on the ratio between the non-navigation and the navigation effective doses.

### Average effective radiation ratio: standard versus navigation protocol

We observed a sixfold increase in the average effective radiation dose between standard protocol sinus CT and 3D navigation which was identical in both children and adults. In the navigation protocol, there also was a statistically significant 20% increase in the adult radiation dose when compared to the radiation dose of the pediatric cohort. To our knowledge, this study is the first to directly compare the effective radiation dose in mSv of standard versus 3D navigation protocol sinus CT scan in an institution, in both an adult and pediatric population. Even if an average effective dose of 2 mSv from a navigation scan remains a small dose and the associated risks for a single exam are negligible, it is of paramount importance to know the true difference in effective radiation doses between non-navigation and navigation sinus CT scans to better raise awareness among otolaryngologists on the irradiation associated with the 3D navigation sinus CT protocols. The younger age of the patient and the possibility of repeated radiological exams for sinusitis or other conditions should prompt the physician to be meticulous in his radiological prescriptions.

### Average effective dose: importance of the mAs

The radiation doses in any specific radiological protocol is determined by the reference mAs. In our institution, for a standard CT scan of the sinus, the reference value was 60 mAs as opposed to the navigation protocol which was 390 mAs. This means that the scanner will set its exposition parameters around these reference values. The larger reference mAs values are associated with a higher definition of the images with less noise. These reference values can be changed according to the level of definition required for adequate navigation. There is a subjective aspect in this decision from the surgeon’s standpoint. Also, the nature of the disease such as polyps may require a higher reference value setting due to the required contrast with the bony structures.

The increases of radiation doses caused by 3D navigation CT protocols of any particular body region has been common knowledge for quite some time [31, 32]. In two different multicenter surveys done in 2009 [32] and 2011 [33], the authors reported respectively a tenfold and 18-fold variation in minimum and maximum doses of radiation for various sinus CT scan imaging protocols. Such variations may be explained by many factors such as the specific parameters and also, the type of scanner. Even though some centers use a unique acquisition protocol, many centers still have a specific 3D navigation protocol and a specific standard CT protocol for sinus CT scans [17].

In our study, 24% (4/17) of pediatric patients and 41% (21/51) of adult patients undergoing 3D navigation sinus CT had already had a sinus CT for the same episode of care. In addition, 3D navigation sinus CT confirmed a surgical indication or was utilized during a surgical procedure in 65% (11/17) of pediatric patients and 49% (25/51) of adult patients. The dilemma persists. On one hand, initial imaging with a 3D navigation protocol may not yield indication for surgery, which results in a non-necessary increase in irradiation for a patient. On the other hand, if one prescribes a standard sinus CT scan which reveals positive findings and criteria for endoscopic sinus surgery, the patient may need a subsequent 3D navigation protocol sinus CT scan with further radiation exposure. This raises a few questions: (1) Is there a clinical pattern that would have better predictability for a positive scan and surgical indication? (2) Is there a regional or seasonal variation in the significance of these patterns? and (3) Are these patterns the same for adults and children? Furthermore, patients with chronic diseases or neoplastic diseases may undergo repeated CT scans during a lifetime.

In our center, the aforementioned data has urged us to work on globally accepted dose-reduction strategies for sinus CT scanning. Among the possible paths, radiation reduction strategies for sinus CT scans have been studied and include adjusting specific scanner parameters [34–36], using iterative reconstruction techniques [37] or using cone-beam technologies [38–40]. We have used eye shielding in the past but our CT scans were inaccurate for navigation. For our specific scanner, X-CARE allows to reduce direct X-ray exposure for the most dose-sensitive body regions, e.g. the breasts, thyroid gland or eye lens through partial scanning. It protects these areas from direct X-ray exposure by lowering the tube current for a certain range of projections. It automatically adjusts the tube current for the remaining projections to prevent deteriorating image quality. Reduced sensitive-area exposure up to 40% was obtained without loss of image quality.

It seems likely that the most effective way to reduce radiation would be to eliminate unnecessary radiologic examinations. With that in mind, using a universal sinus CT protocol that fits both standard and 3D navigation needs may be a way to decrease radiation exposure. Hoxworth and al [17] validated a unique acquisition protocol for both standard and 3D navigation sinus CT scans in their center. In their study, 6187 sinus CT scans were performed, from which 596 were used for image-guided endoscopic sinus surgery. Intra-operative guidance with these CTs were all deemed technically adequate by surgeons. Unfortunately, radiation dose reduction per patient was not significant and translated into an effective dose reduction around 0.8 mSv. Furthermore, no decrease in radiation exposure per CT examination was obtained with their new protocol. Therefore, using a single protocol of this sort brings another kind of dilemma: even if the irradiation per person could be reduced by limiting the number of examinations, patients who do not require surgery would ultimately end up being exposed to higher levels of radiation. Another way to reduce radiation exposure could be to reserve the prescription 3D navigation CTs to physician with a certain area of expertise (OHNS, neurosurgeons, etc.). Judicious prescription of standard sinus CT protocols should also be advocated for primary care physicians as well as other specialists. This could play a role in improving the diagnostic yield and utility of these exams.

Navigation based surgery is a useful tool for the surgeon and has been widely used in sinus surgery [41, 42]. The American Academy of Otolaryngology – Head & Neck Surgery has published a position statement regarding the appropriate indications for computer-guided sinus surgery. These indications include: (1) revision surgery; (2) distorted anatomy (developmental, traumatic or postoperative); (3) extensive sino-nasal polyposis; (4) pathology of the frontal, posterior ethmoid and sphenoid sinuses; (5) disease involving the skull base, orbit, optic nerve or carotid artery; (6) CSF rhinorrhea or other skull base defects and (7) benign or malignant sino-nasal neoplasms [41]. A surgeon’s choice to use 3D image guided surgery technology may rely on these official indications as well as his preference and comfort. Although the use of this technology has demonstrated several advantages, it remains essential to consider the negative effects of increased irradiation on patients [40]. This is even more important for the pediatric population, in which the use of image-guided sinus surgeries is becoming increasingly frequent [5, 8, 10].

Our study has some limitations. First of all, our measurements were obtained from a single CT scanner and a limited number of patients were included. However, because of the considerable difference in irradiation, statistically significant differences were obtained even with our small population. Also, using a single CT device allowed us to have more comparable measurements between the standard and 3D navigation groups. Secondly, radiation doses from 3D navigation protocols can vary greatly. Our scanner and CT protocols are different from other centers. Furthermore, the different navigation system companies still provide different acquisition settings for both standard and 3D navigation sinus CT scans (Table 1).

Our study therefore emphasizes the importance of reviewing one’s own center practices. A patient-oriented approach is suggested including the referring physicians, general practitioners, pediatricians and the attending otolaryngologists to determine the exact indications for navigation and non-navigation sinus scanning and its proper timing. Regarding radiation doses, the technical requirements of the image guidance system must be met including the slice thickness, their overlapping and the minimum field of view. A medical physicist must study the other technical aspects such as the clinical software applications available on the CT scan to minimize irradiation. In cooperation with the physicians, he also must work on optimisation of the acquisition parameters with maintenance of the quality of the images and their accuracy for 3D navigation. A feedback process should be established to monitor each institution’s progress in radioprotection.

## Conclusion

Navigation based surgery is a useful tool for the surgeon and is now widely used for both pediatric and adult patients. In our center on a single scanner, utilization of 3D navigation protocols for sinus CT imaging was associated with a sixfold increase over non-navigation protocols in effective radiation dose. Otolaryngologists should be aware of the possibility of a significant increase in radiation doses for navigation CT and should attempt to decrease the radiation exposure for their patients. A patient-oriented approach is suggested including a review of the prescription practices by referring physicians and otolaryngologists, a close monitoring of the radiological protocols and a feedback process to insure optimal radioprotection.

## Data Availability

We did not seek approval from the research ethics board to share data that was used in this retrospective cohort study. This will be requested if needed.
